# Pathology

**Published:** 1879-04

**Authors:** 


### PATHOLOGY.

DERMOID CYST IN THE CEREBELLUM.—(*Br. Med. Jour.*, Nov. 30, '78.)

At a November meeting of the London Pathological Society, Dr. Irvine showed the brain of a child who was admitted into Charing Cross Hospital for partial loss of power in the lower limbs. She was seven years old, and the only history was that two or three years before she had received a severe blow on the back of the head. Soon after admission, she suffered from convulsions, occurring frequently till her death, three months later. Before death she presented a double internal strabismus and complete paraplegia.

*Post mortem* the dura was adherent to the skull and to the cerebellum. On cutting through it there escaped considerable semi-fluid, pus-like fluid, which was found to be sebaceous matter. In the fluid was found a tuft of hair. The cyst occupied both halves of the cerebellum, and reached down to the cervical part of the cord. The neighboring parts were somewhat inflamed and softened. Dr. Irvine believed the presence of this dermoid substance in the brain could be explained by reference to the embryonic development of the parts.

The specimen was considered unique.

